# Plasma Amino Acid Concentration in Obese Horses with/without Insulin Dysregulation and Laminitis

**DOI:** 10.3390/ani12243580

**Published:** 2022-12-18

**Authors:** Sabita Diana Stoeckle, Detlef Timmermann, Roswitha Merle, Heidrun Gehlen

**Affiliations:** 1 Equine Clinic: Surgery and Radiology, Freie Universitaet Berlin, 14163 Berlin, Germany; 2 Laborservice Onken GmbH, 63584 Gruendau, Germany; 3 Institute for Veterinary Epidemiology and Biostatistics, Freie Universitaet Berlin, 14163 Berlin, Germany

**Keywords:** insulin resistance, equine metabolic syndrome, amino acid, GABA, citrulline, methionine

## Abstract

**Simple Summary:**

Laminitic horses commonly suffer from an endocrine disease, such as equine metabolic syndrome (EMS). Hyperinsulinemia, which is in EMS patients caused by the inability to respond adequately to an oral carbohydrate load, is considered a key factor in the pathogenesis of laminitis. Since insulin also affects protein turnover in the body, the resting plasma amino acid concentrations of obese horses that were presented for a combined glucose insulin test (CGIT), which examines the insulin sensitivity of the tissues, were determined. In total, 25 obese horses and two lean horses with recurrent laminitis underwent a CGIT. Significant differences in the resting concentrations between obese and insulin dysregulated and laminitic (citrulline, GABA, methionine), as well as between insulin dysregulated individuals with and without laminitis (GABA) regarding three amino acids, were determined. This may be an interesting approach, especially for diagnostic testing and possibly also for the feed supplements of horses at risk of developing laminitis. However, further research, including a higher number of cases, is required.

**Abstract:**

Laminitic horses commonly suffer from an endocrine disease such as equine metabolic syndrome. Hyperinsulinemia is considered a key factor in the pathogenesis of laminitis. Since insulin also affects protein turnover in the body, the resting plasma amino acid concentrations of obese horses that were presented for a combined glucose insulin test (CGIT) were determined. In total, 25 obese horses and two lean horses with recurrent laminitis underwent a CGIT. Of these, five were not insulin dysregulated (obese), 14 were insulin dysregulated (ID), and eight were insulin-dysregulated and laminitic (IDL). Significant differences in the resting concentrations between obese and insulin dysregulated and laminitic (citrulline *p* = 0.038, obese: 73.001 ± 12.661 nmol/mL, IDL: 49.194 ± 15.486 nmol/mL; GABA *p* = 0.02, obese: 28.234 ± 3.885 nmol/mL, IDL: 16.697 ± 1.679 nmol/mL; methionine *p* = 0.018, obese: 28.691 ± 5.913 nmol/mL, IDL: 20.143 ± 3.09 nmol/mL) as well as between insulin dysregulated individuals with and without laminitis (GABA *p* < 0.001, ID: 28.169 ± 6.739 nmol/mL) regarding three amino acids were determined. This may be an interesting approach, especially for diagnostic testing and possibly also for the feed supplements of horses at risk of developing laminitis. However, further research, including a higher number of cases, is required.

## 1. Introduction

The development of laminitis in horses is often associated with an endocrine disease (equine metabolic syndrome = EMS; pituitary pars intermedia dysfunction = PPID), in which insulin dysregulation occurs (EMS) or can occur (PPID) [1,2,3]. The suspected pathomechanism of endocrinopathic laminitis is hyperinsulinemia, but the mechanism by which hyperinsulinemia can cause laminitis remains unclear [4]. Based on the results of previous studies, it is suggested that insulin has an effect in the lamellar tissue of the hoof via the insulin-like growth factor 1 receptor (IGF-1R) [5,6,7], and also that insulin can directly trigger laminitis even through mechanisms that have not yet been identified [4]. In addition to its effects on the glucose metabolism, insulin is an important regulator of protein metabolism. Due to an increased utilization of amino acids, hyperinsulinemia can contribute to an increased amino acid and protein turnover, for example in the skin or skeletal muscles [8,9,10,11,12]. In humans and dogs that are suffering from necrotic, wandering erythema and/or superficial necrolytic dermatitis, a reduced concentration of amino acids in the blood was found in some cases. After supplementation with amino acids the clinical picture improved or disappeared [13,14,15,16]. Since hoof horn is an appendix to the epidermis, the changes that occur in laminitis could also be influenced by an altered amino acid concentration. A recently published study in healthy horses showed that horses with euglycemic, hyperinsulinemic clamps for 48 h and glucose infusion for 66 h, had a reduction in blood plasma amino acids and the clinical signs of laminitis [4]. Furthermore, altered postprandial levels of citrulline, histidine, isoleucine, leucine, methionine, ornithine, tyrosine, and valine in horses with EMS, were described previously [17].

Therefore, the aim of our study was to identify differences in fasting amino acid concentrations in obese and insulin dysregulated horses with and without laminitis.

## 2. Materials and Methods

### 2.1. Study Participants

For this prospective study, adult horses and ponies (>3 years old, horses and ponies are both referred to as “horses”) that presented to the Equine Clinic: Surgery and Radiology of the Freie Universität, Berlin, for a combined glucose-insulin test (CGIT) and radiographic evaluation of the distal phalanges (front limbs or both hind and front limbs), were considered for the study. Except for the obvious obesity, the clinical examination of these horses was within normal limits. None of the horses were lame at walk or showed pain at turning. Recorded data for each patient included age, breed, gender, feeding, treatment with pergolide as well as the body condition score (BCS) [18,19]. As confirmed by previous PPID diagnosis, an increased ACTH concentration depending on season and/or a positive TRH stimulation test was defined. Horses were defined as insulin dysregulated/non-insulin dysregulated according to the results of the dynamic tests (see Section 2.2 Dynamic Testing).

The radiographs of the distal phalanges were evaluated by a specialist for equine medicine (Fachtierärztin für Pferde, SDS). Horses were defined as (chronically) laminitic if there was rotation or ventral displacement of the distal phalanx, as reviewed by Thieme et al. [20].

### 2.2. Dynamic Testing

The horses were stabled in the evening before the day on which the CGIT was performed. On the same day as the test was performed, they received an intravenous catheter before starting the CGIT. All horses tolerated the placement of the catheter well, none of them was insubordinate or required sedation. Before dynamic testing, the horses were fasted for 6 h.

The CGIT was performed as described by Eiler et al. [21]. Basal concentrations of glucose and insulin were determined and a sample for endogenous ACTH measurement, as well as amino acid determination collected. For insulin determination, blood was collected in serum tubes (Sarstedt, Nümbrecht, Germany), and for ACTH measurement, in EDTA tubes (Sarstedt, Nümbrecht, Germany). Within 15 min of collection, the blood samples for ACTH measurement were centrifuged and the samples for insulin concentration were centrifuged within 30 min of collection (bot 2000× *g* for 10 min). Plasma and serum were transported cooled to the external laboratory. 

Samples for plasma glucose concentration were collected in a blood collection syringe (BD A-Line, Becton Dickinson AG, Basel, Suisse) and directly evaluated with an automated clinical blood gas analyzer (Cobas b 123, Roche Deutschland Holding GmbH, Grenzach-Wyhlen, Germany).

Then, 150 mg/kg Glucose (Glucose 40% ad us vet., B. Braun Vet., Melsungen, Germany) and 0.1 U insulin/kg bodyweight (Caninsulin^®^ 40 IU/mL, Intervet Deutschland GmbH, Unterschleißheim, Germany) were administered intravenously. Samples for glucose concentration determination were collected at 1, 5, 15, 25, 35, 45, 60, 75, 90, 105, 120, 135, and 150 min. At 45 min, a sample for the stimulated insulin concentration was also collected. If the glucose concentration had returned to the baseline before completion of the 150 min, the test was terminated. Horses were defined as insulin dysregulated if the glucose concentration did not return to baseline within 45 min after glucose and insulin administration, and/or the insulin concentration was greater than 100 µU/mL at the same time point (45 min).

The ACTH and insulin concentrations were determined by Laboklin (Laboklin GmbH and Co. KG, Bad Kissingen, Germany).

### 2.3. Determination of the Amino Acid Concentration

Blood for the amino acid analysis was collected with the baseline samples of the CGIT in EDTA tubes (Sarstedt, Nümbrecht, Germany). Within 15 min of collection, the samples were centrifuged at 2000× *g* for 10 min and the blood plasma frozen at −20 °C for the amino acid analysis.

#### 2.3.1. Sample Preparation for the Amino Acid Analysis

The samples were prepared by mixing 400 μL plasma and 400 μL sample dilution solution (Lithium Loading Buffer Kit, Biochrom Ltd., Cambridge, UK) which included the internal standard Norleucine 200 nmol/mL) in an Eppendorf tube. To this, 200 μL 10% (*w/v*) 5-sulfosalicylic acid (SSA) solution for the deproteinization were added and the sample was deposited in the refrigerator for 20 min at 4 °C. Afterwards, the sample was centrifuged (Eppendorf centrifuge 5415 C) at 11,000× *g* for 5 min. The supernatant was centrifuged a second time with a nylon membrane filter, pore size 0.22 µm (Laborservice Onken, Gruendau, Germany) at 11,000× *g* for 2 min.

#### 2.3.2. Amino Acid Analysis

For the quantitative analysis, the free amino acids were determined with the Biochrom 30+ amino acid analyzer (Harvard Bioscience, Holliston, MA, USA). The amino acid analysis method is based on ion exchange chromatography with post column derivatization with Ninhydrin (Lithium Buffer 1-6 Kit and NZ-Ninhydrin Reagent Kit, Biochrom Ltd., Cambridge, UK).

The physiological standard sample (amino acid physiological standard solution, 40 amino acids, Laborservice Onken, Gründau, Germany) with known amino acid concentrations 200 nmol/mL standard was compared with the horse plasma samples using the EZ Chrome elite software (Agilent, Santa Clara, CA, USA).

### 2.4. Statistical Analysis

The statistical analysis was conducted with the IBM^®^ SPSS^®^ Statistics Version 28.0.1 (IBM Deutschland GmbH, Ehningen/Germany).

The horses were assigned to the following groups: obese, but non-insulin dysregulated and non-laminitic (obese); insulin-dysregulated and non-laminitic (ID); and insulin dysregulated and laminitic. (IDL). Before performing statistical tests, the data were tested for normal distribution. 

The means of the normally distributed data were compared using a one-way ANOVA and in case of no homogeneity of variance the Welch test, for non-normally distributed data, the Kruskal-Wallis test was used. For post-hoc testing, the Tukey and the Games-Howell Test were employed.

### 2.5. Ethical Statement

The study was not declared according to the German Animal Welfare law §8.1 since all samples were taken as a part of a routine clinical examination. Written owners’ consent to involve their horses in the study was obtained during the admission process at the clinic.

## 3. Results

### 3.1. Study Participants

In total, 12 mares and 15 geldings suspected to suffer from insulin dysregulation were presented to the clinic. Two of the equids were not obese but were tested due to recurrent laminitis. One of these two horses was also treated with pergolide. Of the 27 horses presented for dynamic testing, five were not insulin dysregulated (obese), 14 were insulin dysregulated (ID), and eight were insulin-dysregulated and laminitic (IDL). All laminitic horses were insulin dysregulated.

Included equids were mostly ponies; only five were of other breed (Appaloosa (2), Arabian cross, Warmblood, Andalusian horse).

The equids were aged 3–22 years, and horses being IDL (16.63 +/− 2.32 years) were significantly older than obese individuals (9.8 +/− 2.95 years, *p* = 0.008, Games Howell Test). There was no significant difference regarding weight (345.93 +/− 155.28 kg, *p* = 0.716, ANOVA), BCS (8.04 +/− 0.76, *p* = 0.104), and gender (*p* = 0.103) between the groups. All horses, except for one (IDL), that also received a mineral supplement, were kept on a hay diet exclusively. Except for one horse that received pergolide (IDL) the horses did not receive any medications. Demographic data of the study groups are displayed in Table 1.

### 3.2. Endocrine Testing

There was no significant difference in the baseline blood glucose concentration between the groups (*p* = 0.507, ANOVA), but ID and IDL horses required significantly more time for the blood glucose concentration to return to baseline than obese horses (obese: 35.0 +/− 10 min, ID: 121.3 +/− 31.6 min, IDL: 150.63 +/− 0.5 min; obese vs ID *p* < 0.001, obese vs. IDL *p* < 0.001, Tukey test). Furthermore, the blood glucose concentration was significantly longer elevated in IDL than in ID horses (*p* = 0.026, Tukey test).

The resting insulin concentration was significantly higher in ID (7.08 +/− 3.75 µU/mL) and IDL (15.24 +/− 9.38 µU/mL) horses than in obese horses (obese: 2.5 +/− 0.93 µU/mL; obese vs. ID *p* = 0.002, obese vs. IDL *p* = 0.015, Games Howell test). The stimulated insulin concentration was unavailable for three horses: in two obese horses, the test was cancelled after 25 min since the baseline blood glucose concentration was reached and in one IDL horse the sample was lost on the way to the laboratory. The stimulated insulin concentration was significantly higher in ID (51.83 +/− 22.49 µU/mL *p* = 0.011, Games Howell test) and IDL (144.73 +/− 84.6 µU/mL, *p* = 0.017 Games Howell Test) than in obese horses (18.31 +/− 9.93 µU/mL), but there was no significant difference between ID and IDL (*p* = 0.061, Games Howell test). There was no significant difference regarding the ACTH concentration between the groups (Kruskal Wallis test), but three horses (2 IDL, 1 obese) had resting ACTH concentrations above 100 pg/mL. These horses were not treated with pergolide. Concentrations of ACTH and insulin of the study groups are displayed in Table 2.

### 3.3. Amino Acid Concentrations

Mean and standard deviations of the normally distributed amino acid concentrations as well as median, maximum and minimum of the non-normally distributed amino acid concentrations are displayed in Table 3 and Table 4. Furthermore, the count of available samples and the *p*-value were included into the tables as well.

With ANOVA, significant group differences were identified for citrulline (*p* = 0.038), gamma-aminobutyric acid (GABA, *p* < 0.001), and methionine (*p* = 0.019). For post-hoc testing, the Tukey and the Games Howell tests were employed. 

The plasma citrulline concentration was significantly lower in IDL than in obese horses (*p* = 0.038), there was no significant difference between obese and ID (*p* = 0.071) as well as ID and IDL (*p* = 0.789).

The plasma GABA concentration was significantly higher in obese (*p* = 0.002) and ID horses (*p* < 0.001) when compared to the IDL group. However, there was no significant difference between the obese and ID group (*p* = 1).

Methionine was significantly higher concentrated in the plasma of obese individuals when compared to IDL patients (*p* = 0.018). Significant differences between obese and horses suffering from ID (*p* = 0.441) as well as between the ID and IDL group (*p* = 0.067) were not detected.

## 4. Discussion

This study describes the resting amino acid concentration of 25 obese horses and two lean horses with recurrent laminitis that underwent a CGIT. Of these 27 horses, five were not insulin dysregulated (obese), 14 were insulin dysregulated (ID), and eight were insulin-dysregulated and laminitic (IDL). Significant differences in the resting concentrations between obese and insulin dysregulated and laminitic (citrulline *p* = 0.038, obese: 73.001 ± 12.661 nmol/mL, IDL: 49.194 ± 15.486 nmol/mL; GABA *p* = 0.02, obese: 28.234 ± 3.885 nmol/mL, IDL: 16.697 ± 1.679 nmol/mL; methionine *p* = 0.018, obese: 28.691 ± 5.913 nmol/mL, IDL: 20.143 ± 3.09 nmol/mL) as well as between insulin dysregulated individuals with and without laminitis (GABA *p* < 0.001, ID: 28.169 ± 6.739 nmol/mL) regarding three amino acids were determined.

The plasma amino acid profile may be affected by the type of feeding the horses receive. All horses were exclusively fed with hay except for one that received a mineral supplement additionally. However, differences in the nutrient content of hay were reported previously [22,23]. Since all horses were kept off feed for six hours prior to testing, the influence of the feeding is not considered as relevant.

Additionally, three horses had laboratory signs for pituitary pars intermedia dysfunction (PPID) in which the increased endogenous glucocorticoid concentrations are known to be associated with systemic insulin resistance [24,25,26,27,28,29] and thus may have influenced the amino acid profile additionally [30,31]. One further horse was treated with pergolide which may have additionally influenced the concentrations of the amino acids [30].

An optimal study would have compared larger groups of horses that were kept under the same conditions for a few weeks and that did not show signs of additional endocrinopathies.

Stokes et al. reported significant changes of the plasma amino acid concentration in an experimentally produced hyperinsulinemia [4]. However, they found slightly different changes during the euglycemic hyperinsulinemic clamp compared to the prolonged glucose infusion [4]. Furthermore, they found a significant decrease in 15 (prolonged glucose infusion) and 19 (euglycemic hyperinsulinemic clamp) of the 20 amino acids determined in the plasma [4], whereas in this study, only three amino acids (citrulline, GABA, methionine), of which not all are proteogenic, had significantly different concentrations between obese and insulin dysregulated and laminitic (citrulline, GABA, methionine) as well as between insulin dysregulated individuals with and without laminitis (GABA). This may be attributed to the fact, that resting amino acid concentrations were measured and none of the horses had a resting hyperinsulinemia. However, in both the euglycemic hyperinsulinemic clamp and the prolonged glucose infusion, the concentration of methionine was one of the amino acids that showed a marked decrease during hyperinsulinemia [4] and methionine was also one of the amino acids that showed a significant difference between insulin dysregulated non-laminitic and laminitic animals. Since none of the horses showed a resting hyperinsulinemia, less changes were observed in this study. Contrasting to this, higher postprandial concentrations of citrulline and methionine after a high protein meal were described in horses suffering from EMS when compared to healthy horses [17]. It may have been interesting to examine the amino acid concentrations after 45 min, at least in this cohort with marked increases in the insulin concentration after stimulation.

Methionine, the amino acid that showed marked decreases during induced hyperinsulinemia [4] and was significantly less concentrated in the plasma of insulin dysregulated and laminitic horses than in the plasma of obese horses, is an essential proteinogenic amino acid [32]. Furthermore, methionine as well as cysteine are a part of the glutathione metabolism, which is the major antioxidant in mammalian cells [33]. In humans, a nutritional methionine deficit was associated with diseases such as toxemia, childhood rheumatic fever, muscle paralysis, hair loss, depression, schizophrenia, Parkinson’s disease, liver deterioration, and impaired growth [34]. In healthy lactating cows, supplementation with methionine led to an increase hoof horn growth rate, but also to alterations of the amino acid profile of the hoof horn. Supplemented cows had less cysteine and proline but greater percentages of methionine, lysine, tyrosine, and glutamic acid levels in their hoof horn. The authors suggested that this may be caused by a decrease of disulfide bonding in the hoof tissues [35]. Another study in heifers during the first 13 weeks of lactation found increased growth rates but unchanged wear rates during methionine supplementation which led to conformational changes of the hoof [36]. Other authors reported a significant decline of heel erosions, sole avulsions, and the resolution of all the white line hemorrhages [37]. However, methionine was claimed to be the most toxic amino acid in relation to growth in animals [38]. Contrasting to this, a review on methionine toxicity in humans concluded that serious methionine toxicity only occurs only at very high levels of intake [39]. In animals, methionine and cysteine toxicity were associated with consumption at levels five times greater than required [40] and the free sulfhydryl group of cysteine was assumed to be the cause of toxicity in chicks [41]. However, the mechanism of toxicity is not completely understood [42]. A study examining methionine supplementation in weanling Quarter horses (basal: 0.2% methionine, basal + 0.03% methionine: 0.23% methionine, basal + 0.07% methionine: 0.27 methionine, basal + 0.11% methionine: 0.3% methionine) suggested that the methionine requirements for growing Quarter horses may fall between 0.23% and 0.31% methionine [42]. Requirements for insulin-dysregulated horses have not been published so far. This may be interesting for further research especially since beside Stokes et al. [4] and a study by Kenéz and colleagues identified significantly lower methionine in addition to lower trans–4 hydroxyproline levels in insulin-dysregulated, compared to insulin–sensitive horses [43].

GABA is a non-proteogenic amino acid which acts as an inhibitory neurotransmitter in mammalian neural tissues [44]. However, recent research on wound healing in rats suggests an anti-inflammatory and fibroblast proliferation stimulating role of GABA. In this study, GABA treatment was effective in accelerating the healing process [45]. Additionally, a study in humans identified an improvement of the skin elasticity in humans of GABA by regulating type I collagen expression [46]. As commonly known, hoof horn is the appendix to the epidermis, at which the damage during endocrinopathic laminitis occurs. In the case of a GABA deficiency during hyperinsulinemia, the healing of the damage that occurred during hyperinsulinemia might be delayed. This seems to be an interesting subject for future studies. As GABA, citrulline is a non-proteogenic amino acid as well, which acts as an intermediate in the metabolite in the ureagenesis [47,48]. Since citrulline is almost exclusively metabolized by the small intestine, the plasma citrulline concentration is considered as a biomarker of the functional small intestinal bowel mass [49,50]. Furthermore, citrulline is a functional biomarker in renal failure since the kidney is the only organ that metabolizes citrulline into arginine [51], which is a major regulator of vascular tone [52,53,54]. Since in some cells arginine can be recycled from citrulline, it can act as a precursor for arginine [55] and may therefore be of importance for the nitric oxide (NO) metabolism and regulation [56]. NO-mediated vasodilation and GLUT 4 translocation are initiated by stimulation of insulin receptors in healthy horses [57]. In insulin resistance, this pathway may be blocked and therefore the alternative mitogen-activated protein kinase pathway activated. This leads to endothelin 1-mediated vasoconstriction, the upregulation of cellular adhesion molecules, and mitogenesis [57]. In this study, insulin dysregulated laminitic horses had significantly lower citrulline concentrations than obese animals. Furthermore, a study focusing on the citrulline concentration in horses with gastrointestinal disease found lower citrulline concentrations in horses developing laminitis compared to those who did not [58]. The clinical relevance of this finding remains to be elucidated. Clearly, further studies are required to examine amino acid concentrations as a marker in equine endocrinopathic disease, or as a potential supplement for (previously) laminitic horses.

## 5. Conclusions

There are different resting concentrations between obese and insulin dysregulated and laminitic (citrulline, GABA, methionine) as well as between insulin dysregulated individuals with and without laminitis (GABA). This may be an interesting approach especially for diagnostic testing and possibly also for feed supplements of horses at risk of developing laminitis. However, further research including a higher number of cases is required.

## Figures and Tables

**Table 1:** Demographic data of study participants (mean +/− standard deviation or absolute numbers of horses).

	Obese	Insulin Dysregulated	Insulin Dysregulated and Laminitic
Age (years)	9.80 ± 2.95	14.14 ± 5.11	16.63 ± 2.32
Weight (kg)	294.0 ± 167.6	353.6 ± 137.1	365.0 ± 190.2
Body condition score	8.0 ± 0.0	8.3 ± 0.5	7.6 ± 1.1
Previous diagnosis of PPID	0	0	1
Treatment with pergolide	0	0	1

**Table 2:** ACTH and insulin concentration in obese, insulin dysregulated, and insulin dysregulated laminitic horses (median (minimum-maximum) or mean +/− standard deviation).

	Obese	Insulin Dysregulated	Insulin Dysregulated and Laminitic
ACTH (<30 pg/mL)	19 (12.5–196)	21.9 (10.1–80.8)	16.7 (9.8–963)
Insulin at 0 min (<20 µU/mL)	2.5 +/− 0.93 µU/mL;	7.08 +/− 3.75 µU/mL	15.24 +/− 9.38 µU/mL
Insulin at 45 min (<100 µU/mL)	18.31 +/− 9.93 µU/mL	51.83 +/− 22.49 µU/ml	144.73 +/− 84.6 µU/mL

**Table 3:** Mean and standard deviations of the normally distributed amino acid concentrations (nmol/mL).

Amino Acid		Obese	ID	IDL	*p* (ANOVA)
1-Methyl-Histidine	Mean	23.986	19.414	17.147	0.335
	Standard deviation	10.613	8.663	5.062	
	Available Samples	27	27	27	
Alanine	Mean	206.175	226.394	223.001	0.789
	Standard deviation	69.407	46.584	64.663	
	Available Samples	27	27	27	
Arginine	Mean	73.388	65.889	69.635	0.772
	Standard deviation	18.05	17.323	20.641	
	Available Samples	27	27	27	
Asparagine	Mean	19,858	18,167	20,147	0.726
	Standard deviation	7.009	4.08	8.183	
	Available Samples	27	27	27	
Citrulline	Mean	73.001	53.724	49.194	0.038
	Standard deviation	12.661	16.95	15.486	
	Available Samples	27	27	27	
GABA (Gamma-aminobutyric acid)	Mean	28.234	28.169	16.697	<0.001
	Standard deviation	3.885	6.739	1.679	
	Available Samples	27	27	27	
Glutamine	Mean	263.382	254.664	237.805	0.728
	Standard deviation	61.853	50.585	74.643	
	Available Samples	27	27	27	
Glutamic acid	Mean	16.292	18.53	25.391	0.116
	Standard deviation	2481	6956	12,126	
	Available Samples	27	27	27	
Glycine	Mean	43.641	393.742	369.839	0.669
	Standard deviation	84.764	152.069	10.065	
	Available Samples	27	27	27	
Histidine	Mean	73.297	76.231	71.124	0.314
	Standard deviation	10.55	7.2	5.819	
	Available Samples	27	27	27	
Lysine	Mean	70.1	68.014	80.389	0.363
	Standard deviation	17.088	21.831	15.858	
	Available Samples	27	27	27	
Methionine	Mean	28.691	25.617	20.143	**0.019**
	Standard deviation	5.913	4.862	3.09	
	Available Samples	27	27	25	
Ornithine	Mean	48.718	49.153	48.782	0.997
	Standard deviation	10.915	15.553	11.795	
	Available Samples	27	27	27	
Phenylalanine	Mean	52.286	53.543	56.846	0.491
	Standard deviation	12.96	6.437	3.833	
	Available Samples	27	27	27	
Proline	Mean	61.044	57.062	61.151	0.834
	Standard deviation	20.69	10.817	23.946	
	Available Samples	27	27	27	
Serine	Mean	181.459	214.725	229.933	0.420
	Standard deviation	45.655	66.886	66.504	
	Available Samples	27	27	27	
Taurine	Mean	39576	36.345	33.037	0.674
	Standard deviation	5.812	15.998	9.218	
	Available Samples	27	27	27	
Tryptophan	Mean	64.037	64.699	72.756	0.308
	Standard deviation	11.293	12.67	12.661	
	Available Samples	27	27	27	
Valine	Mean	160.249	163.951	190.369	0.274
	Standard deviation	24.689	39.447	46.052	
	Available Samples	27	27	27	

**Table 4:** Median, minimum, and maximum of the non-normally distributed amino acid concentrations (nmol/mL).

Amino Acid		Obese	ID	IDL	*p* (Kruskal Wallis Test)
Isoleucine	Median	44.325	59.348	65.526	0.084
	Maximum	66.544	100.782	84.085	
	Minimum	41.413	35.487	57.173	
	Available Samples	27	27	26	
Leucine	Median	77.476	98.757	120.116	0.078
	Maximum	128.293	123.473	143,189	
	Minimum	71.808	69.43	60.7	
	Available Samples	27	27	27	
Threonine	Median	117.291	81.244	67.396	0.59
	Maximum	149.826	170.211	212.1	
	Minimum	50.631	58.425	39.823	
	Available Samples	27	27	27	
Tyrosine	Median	70.656	62.53	58.03	0.806
	Maximum	76.974	74.186	84.769	
	Minimum	35.127	43.782	50.1	
	Available Samples	27	27	27	

## Data Availability

Additional data can be obtained upon request from the corresponding author.
